# Longitudinal Evaluation of Dysarthria Progression in Patients with Parkinson’s Disease

**DOI:** 10.3390/diagnostics16050683

**Published:** 2026-02-26

**Authors:** Wilmar Alesander Vásquez-Barrientos, Daniel Escobar-Grisales, Cristian David Ríos-Urrego, Juan Rafael Orozco-Arroyave

**Affiliations:** 1 GITA Lab, Faculty of Engineering, University of Antioquia, Medellin 050010, Colombia; alesander.vasquez@udea.edu.co (W.A.V.-B.); daniel.esobar@udea.edu.co (D.E.-G.); cdavid.rios@udea.edu.co (C.D.R.-U.); 2 LME Lab, University of Erlangen, 91058 Erlangen, Germany

**Keywords:** Parkinson’s disease, speech analysis, longitudinal modelling, classification, modified Frenchay Dysarthria assessment

## Abstract

**Background/Objectives:** Automatic evaluation of Parkinson’s disease (PD) progression is an emerging topic that deserves special attention from the research community. Unobtrusive, low-cost technology is essential for monitoring PD patients in remote areas. This paper proposes the use of phonological posteriors to create models that allow the progression of dysarthria level progression to be modelled based on speech recordings. **Methods:** Eighteen Gated Recurrent Units (GRUs) are used to estimate an equal number of phonological classes assigned to each phoneme pronounced in a given recording. Classification models of PD vs. healthy control (HC) subjects are trained with recordings of the PC-GITA corpus. This information is used in a separate corpus, with longitudinal recordings, to evaluate whether the progression of the dysarthria level, according to the modified Frenchay Dysarthria Assessment (mFDA), is related to abnormal production of specific phonemes. **Results:** Strident, dental, pause, back, and continuant phonological classes are the ones that better explain dysarthria level progression within time-frames of at least two years, therefore allowing possible monitoring of disease progression. **Conclusions:** Speech is a low-cost biosignal that can be used to automatically assess PD progression. In particular, this study shows that such an assessment makes it possible to evaluate dysarthria level progression and to find which phonological classes are contributing the most to such a progression. We believe that the findings reported in this paper provide objective evidence about possible abnormalities in broader speech-related processes like respiration, therefore contributing a better understanding of the relationship between speech production patterns and other speech-related processes affected when suffering from PD.

## 1. Introduction

Parkinson’s disease (PD) is a progressive neurodegenerative disorder that affects around 10 million people worldwide [1]. This disease is characterized by different motor and non-motor symptoms, including rigidity, resting tremor, bradykinesia, depression, sleep disorders, and others [2,3].

As the disease progresses, patients exhibit a progressive deterioration of one or several symptoms, which therefore negatively impacts their quality of life. Given that motor and non-motor alterations progress differently among patients, timely, accurate, and constant monitoring is required to make the administration of appropriate/individualized treatment possible [4]. Continuous and unobtrusive monitoring constitute current challenges for all health systems across the globe [5]. Such challenges motivate the research community to develop automatic monitoring tools that allow the characterization of disease progression in a timely and accurate manner, considering different biomarkers. Among the existing biomarkers, speech arises as one of the most promising given the simplicity, unobtrusive nature, and low cost of its monitoring [6].

Speech disorders affect around 89% of PD patients [7,8] and it is known that such alterations appear in the early stages of the disease [9]; therefore, speech analysis is not only a promising biomarker with which to monitor the disease progression, but also to detect it early on [10]. From a clinical perspective, dysarthric speech is characterized by producing slow, weak, and imprecise muscle movements involved in speech production. These patterns can be measured and quantified by different means. For instance, the authors in [11] used a cohort of 63 PD patients and 35 healthy controls and evaluated their vocalic articulation by measuring the area of the vocal triangle, the vowel articulation index, and the Phi index. All these measures are based on the vocalic formants, which provide a simple and well-known method to evaluate articulation in speech. With this simple approach, the authors found that the area of the vocalic triangle is significantly smaller in dysarthric speakers than in healthy controls. With similar approaches, based on vocalic formants extracted from speech spectrograms and also with temporal-related measures, the authors in [12] studied vowel acoustics in dysarthric speakers suffering from different disorders, including ALS, Parkinson’s, Huntington’s, and others. The authors reported that metrics designed to model vowel distinctiveness are more sensitive and specific predictors of dysarthria.

Besides studies on speech production, alterations in other physiological processes like respiration have also been studied. For instance, in [13], the authors evaluated how abnormal respiration in PD patients also affects speech production. The study included a cohort of 55 patients assessed over 15 days. Several motor skills were evaluated, including postural stability, speech production, respiration (at rest and during physical exercises), etc. Patients were grouped according to their dysarthria severity, i.e., mild, moderate, and severe. Their findings indicate that those patients who showed more dysarthria severity also exhibited other comorbidities like abnormal respiration and posture. As a conclusion, the authors claimed that dysarthria and altered respiration are closely related in PD patients and such alterations affect other motor skills important for daily living, like postural stability. Another study [14] involved evaluating intelligibility changes in PD patients (native Spanish speakers) who followed the Lee Silverman Voice Treatment (LSVT-LOUD) therapy. The cohort included a total of 15 patients with PD. According to their results, the authors indicate that those dysarthric patients who performed the therapy improved their intelligibility (perceptually evaluated). From an engineering perspective, most studies are focused on using hand-crafted features like jitter, shimmer, Mel spectra, etc. [15,16,17]. The authors in [18] introduced the use of Gated Recurrent Units to extract phonological posteriors. This approach was later refined in [19] and effectively used to model how PD patients produce the phonemes in continuous speech recordings. The same pattern had been reported a couple of years before in [20], where the authors found that phonological classes like plosives, vowels, and fricatives are the most sensitive to the motor deterioration in PD speech. A similar approach was reported in [21], where the authors showed that phonological posteriors are more effective for detecting PD than classical approaches based on Mel spectra, Mel Frequency Cepstral Coefficients (MFCCs), or the well-known eGeMAPS [22]. A similar finding was reported in a more recent work [23], where the authors not only included classical features like articulation and prosody but also foundation models like Wav2vec to make comparisons w.r.t. the phonetic posteriors.

The reviewed state of the art suggests that most works have been focused on classifying PD patients vs. HC subjects; however, longitudinal evaluation remains under-explored. One of the reasons for this is due to the fact that it is hard to get access to collect speech recordings of PD patients, and such a difficulty is maximized when the patient is requested to be recorded several times within a given period of time. The work of [24] constitutes a seminal study in this direction. The authors evaluated changes in the speech production of 80 PD patients and 60 HC subjects within a time-frame of up to 12 months. The main result indicated a significant deterioration in speech quality, especially in speech velocity and articulation. Following the previous idea, the authors in [25] introduced the use of Gaussian Mixture Models (GMMs) to automatically model the level of dysarthria in a set with 7 patients who were recorded a total of 16 times between 2012 and 2016. The collection process was involved four recordings taken during the same day, every 2 h. The time between each recording session was around one year. The main conclusion of the study was that it is possible to accurately trace the dysarthria level of PD patients longitudinally.

After reviewing the existing literature, it is found that some researchers have addressed the topic considering standard and (to some extent) sophisticated speech modelling methodologies. However, there is one gap that still needs attention: the use of methods that allow clinical interpretability is still an open question. Building on our previous studies, where phonetic posteriors are introduced as a promising tool to model speech production in PD patients [19,26], in this paper, we analyse how different phonological classes change while the disease is progressing.

## 2. Data Collection and Annotation

Two corpora were considered for this study. PC-GITA [27] and a set with 16 speakers who were recorded longitudinally over three years (once per year). PC-GITA is used exclusively to generate the models that extract and “measure” each phonological posterior, and the longitudinal corpus is used to test the suitability of the proposed approach. Theses two sets of speakers are independent. In this study, we decided to use only the recordings corresponding to monologue and read text. For the first case, participants were asked to talk about the activities of their daily routine. This task resulted in audio files with an average duration of 82 s. For the second task, the instruction consisted of reading aloud a phonetically balanced text. This resulted in audio files with an average duration of 16 s. In both cases, the files were not segmented; this means that we did not select specific portions of the recordings but rather took them as complete. We were able to do this because the duration of the audio files was very similar among recording sessions. In any case, to avoid any possible bias due to recording durations, all our computations were averaged across speech frames of 25 ms, as indicated in [18]. All recordings were down-sampled to 16 KHz. The clinical and demographic information of these two groups are presented below.

### 2.1. PC-GITA

This corpus was originally introduced in [27]. The participants were recorded in Medellín, Colombia, in a sound-proof booth, with a professional audio setting and with a sampling frequency of 44.1 KHz. The cohort includes a total of 100 speakers, 50 with PD and 50 HC; all were native speakers of Colombian Spanish. The neurological state of all patients was evaluated by an expert neurologist according to the MDS-UPDRS-III scale (Movement Disorder Society—Unified Parkinson’s Disease Rating Scale, Part III) [28]. PD patients and HC subjects were matched by age and gender. For this work, we only considered two speech tasks, namely monologue and read text. Clinical and demographic details are presented in Table 1.

### 2.2. Longitudinal Corpus

This corpus includes speech recordings of 16 PD patients who are native speakers of Colombian Spanish. Each patient was recorded once per year over the course of three years. Unlike the PC-GITA group, the recordings in this corpus were not collected in a sound-proof booth but in more realistic scenarios, i.e., closer to those that exist in real clinical settings. To avoid possible bias due to acoustic conditions changes, the same recording setting was used in all recordings. Additionally, although acoustic conditions might have changed over time, technicians tried to keep those conditions as similar as possible for all recordings. Besides the recording procedure, all recordings were considered by an expert phoniatrician, who administered the modified Frenchay Disarthria Assessment scale (mFDA), which was introduced in [29] as a clinically suitable way to assess the dysarthria level of PD patients exclusively relying on speech recordings that include speech tasks like read text, sustained vowels, monologue, and diadochokinetic tasks (e.g., rapid repetition of syllables like /pa-ta-ka/). The clinical and demographic information for this cohort is included in Table 2.

### 2.3. Evaluation of Dysarthria Level According to the mFDA Scale

Besides the above-mentioned clinical and neurological evaluations, recordings of all participants in this study were labelled by three expert Speech & Language (S&L) therapists according to the mFDA scale. The inter-rater reliability among the phoniatricians was 0.75, which was computed by calculating the average Spearman’s correlation between all possible pairs of raters [29]. The final label used for this study was taken as the median across the three S&L experts. The mFDA scale consists of 13 items grouped into 7 aspects of speech production, namely respiration, lip movement, palate/velum movement, larynx, tongue, monotonicity, and intelligibility. Each item ranges from 0 (healthy) to 4 (totally impaired). Therefore, the total range of the scale is from 0 to 52 [29]. Since the mFDA scale can be administered based on speech recordings, without requiring the patient to visit the clinic, all participants (PD and HC) were labelled. The reader can see the paper [29] for further details of the scale. The labelling process within the recordings of the PC-GITA corpus yielded a mean value of 8.5 ± 7.4 for the HC group and 28.8 ± 8.3 for the PD group. The results of the labelling process of the three sessions in the longitudinal corpus are shown in Table 3. Similarly, the average values of mFDA labels across the recording sessions are shown in Table 4.

**Figure 1:**
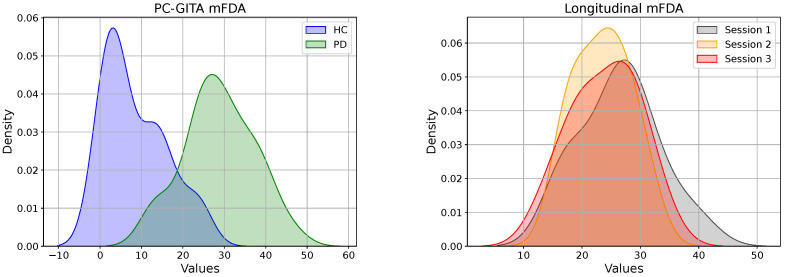
Distributions of the mFDA scores assigned by S&L therapists to the PC-GITA (**left**) and the longitudinal corpora (**right**).

Figure 1 shows the distribution of the mFDA values assigned by the S&L experts to recordings of the PC-GITA (left) and longitudinal corpora (right). Notice that there is a clear separation between most PD and HC speakers in the PC-GITA group. However, there is overlap in some cases because PD does not affect speech production equally across patients, and also because some HC speakers might not exhibit completely healthy speech. Notice also that, on the right-hand side, the first impression is that almost all patients exhibited similar dysarthria levels in the three recording sessions of the longitudinal corpus. This demonstrates the complexity and relevance of the problem we are tackling in this work.

## 3. Methodology

**Figure 2:**
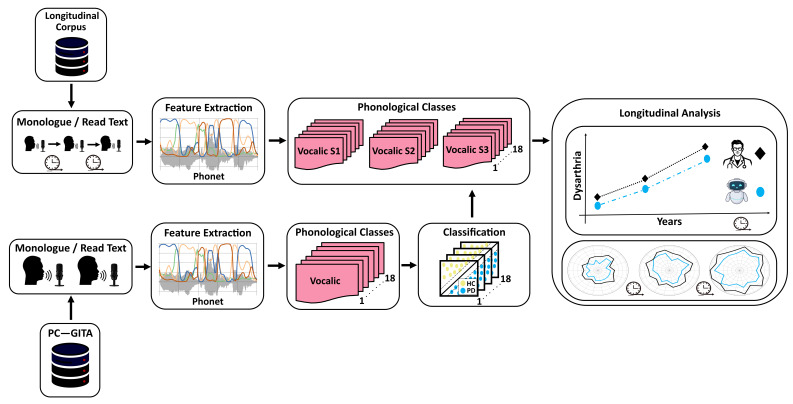
General methodology. Phonological features are computed from the speech recordings of PC-GITA. Such features are grouped according to 18 phonological classes, and an equal number of classification models are trained (one per phonological class). The same phonological features and corresponding classes are extracted from the longitudinal corpus. The classifiers optimized with PC-GITA are used to evaluate the longitudinal recordings. Resulting classification scores are used to map the corresponding mFDA scores to model dysarthria progression across the different recording sessions. Comparisons are made via radar plots, whose axes are the phonological classes, therefore enabling direct interpretation.

Figure 2 illustrates the general methodology proposed in this study. Monologue and read-text recordings of the PC-GITA corpus are used to create phonological models with the Phonet module, originally introduced in [18]. It provides values for the phonological posteriors associated with each phonological class for a given phoneme. Therefore, feature vectors are created by grouping posteriors according to their phonological class. Each feature vector is used to train individual classifiers per phonological class. Resulting models are evaluated on the PC-GITA recordings following a 10-fold cross validation strategy. Optimal meta-parameters of the classifiers are stored to be used later in the evaluation of longitudinal recordings. Notice that no further optimization is performed in this step. The final aim is to evaluate which phonological classes are more sensitive to disease progression, which ultimately would provide additional information to the expert neurologist and speech/language therapist to make appropriate decisions regarding medication and therapy updates. Details of each stage in the methodology are provided in the next subsections.

### 3.1. Phonological Representation

Phonological representations are created with Phonet, which is based on bi-directional Gated Recurrent Units (GRUs) that were trained with 17 h of Latin American Spanish recordings. Notice that the outcome of such a model was named in the literature as Phonemic Identifiability [26]. Among several properties, it demonstrated a great sensitivity to model cognitive decline in PD patients. Phonet constitutes one of the modules of the Disvoice toolkit (https://github.com/jcvasquezc/Disvoice (accessed on 22 February 2026)). The outcome includes a total of 21 phonemes which are grouped into 18 phonological classes, as described in Table 5. The overall accuracy of Phonet in modelling the 18 phonological classes is 86.6% [18]. Notice that silence is considered as one of the classes.

Phonological posteriors are calculated in segments of 80 ms length with an overlap of 40 ms. Therefore, each audio recording has a variable length representation. To create fixed-length vectors, six statistical functionals are computed, namely mean, standard deviation, maximum, minimum, skewness, and kurtosis. Ultimately, each audio is represented with a total of 18 6-dimensional feature vectors, one per phonological class.

### 3.2. Automatic Classification of PD vs. HC Subjects

After creating phonological representations for all speakers in the PC-GITA corpus, the resulting feature vectors were used to train a Support Vector Machine (SVM) classifier. Three kernels were evaluated: linear (Lin), sigmoid (Sig), and Gaussian with a radial-basis function (RBF). Optimization of the corresponding meta-parameters was performed following a speaker-independent 10-fold nested and stratified cross-validation. Notice that after this stage, there will be 18 independent models, one per phonological class. Each model is given by its corresponding optimal parameters: *C* for the case of linear and sigmoid kernels or *C* and γ for the Gaussian and its support vectors.

### 3.3. Longitudinal Analysis

Phonological posteriors are also extracted from recordings of the Longitudinal corpus. Thus, speakers in this stage are also represented as 18-dimensional feature vectors. The last stage in the methodology consists of taking those 18 optimal classification models found in the previous stage and computing their corresponding output, i.e., classification scores, for the recordings in the Longitudinal corpus. Since no further optimization was performed at this stage, the Longitudinal corpus constitutes an independent test set, which besides being recorded in realistic conditions, allows the evaluation of disease progression over three years. It is important to stress the fact that none of the speakers in this corpus were included in the previous stage (the one based on PC-GITA).

## 4. Experiments and Results

Two main experiments were performed in this work: (i) automatic classification of PD vs. HC subjects considering the phonological posteriors extracted with the Phonet module and within the context of PC-GITA and the (ii) automatic evaluation of dysarthria level progression, also based on the corresponding phonological posterior, within the context of the Longitudinal corpus. Two speech tasks were considered separately in both experiments: monologue and read texts.

### 4.1. Classification of PD vs. and HC

There was one independent classifier per phonological class. Thus, a total of 18 classification results were found per speech task. This experiment allowed us to evaluate which are the most sensitive phonological classes prior to continuing with the longitudinal evaluation. All results per phonological class are included in Table 6. Due to space limitations, only kernels associated with the best results are shown. The upper part of the table indicates results of the monologue while the bottom part includes those of the read texts. In both cases, the average performance across phonological classes is around 65% accuracy, which despite not being very high, provides the additional advantage of being clinically interpretable. Values and corresponding phonological classes with accuracies above 70% are highlighted in bold.

Notice that both speech tasks (monologue and read text) indicate that among the most discriminative phonological classes are **back** and **open**. This indicates that the movement and control of articulators involved in the production of vowel phonemes, e.g., tongue, jaw, and lips, play a crucial role in the evaluation of speech production. Other classes that were shown to be relevant for automatic discrimination were **flap** and **lateral**, which implies that the abnormal production of the /l/ and /R/ phonemes is also characteristic of Parkinson’s speech. Notice that the production of these phonemes also requires proper control of the tongue, as in the case of the above-mentioned classes. Finally, to some extent, these observations can be summarized by the fact that the **consonantal** class yielded good classification results when the read texts were considered. Notice that this phonological class includes phonemes like /m/, /n/, /l/, /t/, /p/, and /k/, which require the accurate control of the tongue, velum, and lips.

Based on the aforementioned results, the next experiment considers the evaluation of disease progression considering the phonological posteriors. The main hypothesis is that there are certain phonological classes that allow disease progression to be observed more accurately, and those phonemes are such that they require the control of specific articulators.

### 4.2. Automatic Dysarthria Level Progression Evaluation

This experiment considers speakers of the longitudinal corpus as the test set exclusively. This means that models resulting from Section 4.1 are taken as they are, without any further optimization of meta-parameters. One classification model is created from each phonological class. Dysarthria level progression is evaluated in two scenarios: (i) on the set of 16 patients in the longitudinal corpus and (ii) on a subset of 7 patients who showed effective dysarthria progression according to the mFDA values in the longitudinal corpus. Effective progression between session 1 and session 3 was considered as the inclusion criterion.

#### 4.2.1. Sensitivity Analysis of the Longitudinal Recordings

**Figure 3:**
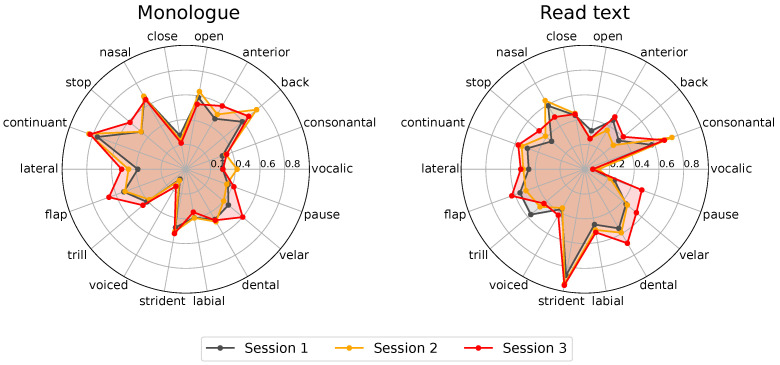
Classification scores obtained after evaluating the three recording sessions of the longitudinal corpus. Each axis represents the obtained classification score for corresponding phonological classes.

This experiment intends to find which phonological classes best model PD progression, considering the three recording sessions of the longitudinal corpus. Figure 3 shows two radar plots that include the classification scores per phonological class obtained from monologues and read texts. Only results from the 16 patients of the longitudinal corpus are considered. This is the reason why we associate this analysis with a sensitivity measurement. The result of the analysis is summarized in Figure 3. Three different plots are included per speech task: one per recording session. Notice that both figures show minimal changes in different phonological classes across recording sessions. Notice also that, by comparing sessions 1, 2, and 3, we are implicitly considering a time span of 12 months, which may not be long enough to observe changes in the dysarthria level of PD patients. According to [24], changes in the motor skills of PD patients are not clearly observable within a year.

In the context of SVM classifiers, classification scores are directly associated with the distance of the given sample to the separating hyperplane. Therefore, through this analysis we intend to study the relationship between those scores and the mFDA values of corresponding samples. The main assumption is that the larger the classification score, the more advanced the dysarthria level of the corresponding patient. This assumption is supported by the fact that, if the dysarthria level of a given patient increases, the classifier will be more confident regarding its belonging to a particular class, and will therefore locate that particular speaker at a more distant point in the latent space, resulting in a larger distance. Of course, the opposite can also happen, i.e., one patient can be located far away from the separating hyperplane in the first recording session, and get closer in the second or in the third recording session. This could happen due to medication effects or mood changes, but these phenomena are beyond the scope of this paper.

One possible way of objectively evaluating whether phonological posteriors are modelling disease progression across the three recording sessions is to measure the area of each radar-plot resulting from each recording session. Table 7 shows the results of such area computations. Notice that there is a clear increase in the area of session 1 vs. session 3 for both speech tasks, monologue and read text. However, although the areas show the progression of dysarthria level, it is hard to establish which are the phonological classes that best explain such progression. For this reason, we decided to focus on modelling only those patients for which the mFDA labels evidenced effective dysarthria progression.

#### 4.2.2. Modelling of Effective Dysarthria Level Progression

**Figure 4:**
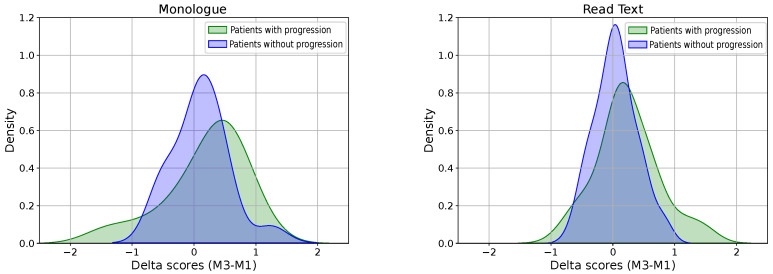
Classification score distributions obtained for the set with seven patients who showed dysarthria level progression between session 1 and session 3 according to the mFDA values (**left**) vs. no progression (**right**).

The results of the labelling process for the longitudinal corpus according to the mFDA scale are indicated in Table 3. As mentioned above, twelve months may be a very short period of time to observe objective changes in speech production patterns. One of the main complexities of PD is the fact that it does not progress the same way for all patients. This makes its modelling a real challenge, regardless of the bio-signal, e.g., speech, gait, handwriting, facial expression, etc. In the case of this work, based on the labels of the mFDA scale, we observed that not all patients showed dysarthria progression from session 1 to session 3. Given this scenario, we decided to take a subset of patients, considering only those who showed progression between these two sessions. After performing this filtering, a total of seven patients from the original sixteen were selected. These patients are highlighted in bold in Table 3. The classification scores distribution obtained for patients in session 3–session 1 is illustrated in Figure 4. Monologue and read text are included on the left and right side plots, respectively. Distributions in green represent the group of patient who showed dysarthria level progression, and distributions in blue represent those patients who did not show progression. Notice that in both cases, the group of patients who showed progression are slightly shifted to the right.

**Figure 5:**
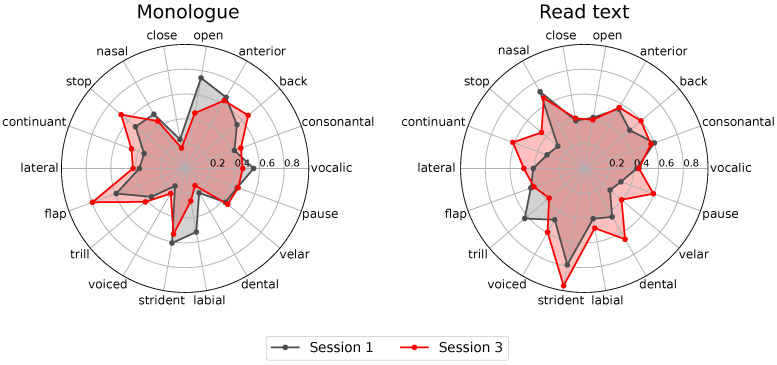
Radar plots of the classification scores obtained per phonological class for the set with seven patients who showed dysarthria progression between session 1 and session 3 according to the mFDA values. Classification scores are indicated per phonological class obtained with the monologue (left) and read text (right) speech tasks. Recordings of session 1 (black) and session 3 (red) are indicated for the analysis.

Once the subset of speakers was created, we repeated the previous processes and created the radar plots to analyse the behaviour of specific phonological classes. The result is shown in Figure 5.

Similarly to the analysis performed for the complete longitudinal cohort, dysarthria progression was objectively measured according to the areas of the radar plots per session. The results are included in Table 8. Notice that these more specific analyses show that the area increase in the case of monologues is minimal, therefore inconclusive regarding specific phonological classes. Conversely, for read text, the area indicated an increase from 0.6695 to 0.8858 (36.8% of increase). Notice that in this experiment, we were focused on those patients who showed dysarthria progression according to a clinical criterion (mFDA values). If this had not been the basis of our consideration, we would have only considered the results in Figure 3 and Table 7. This would have masked the patterns that we can observe when focusing only on these seven patients. For instance, when observing the radar plots in Figure 5, we can conclude that strident, dental, pause, back, and continuant phonological classes are the ones that explain such an increase. Based on the equivalence between phonological classes and phonemes indicated in Table 5, we observe that what predominantly models dysarthria level progression is the control of the *lips* (phoneme /f/)) and *tongue* (phonemes /t/, /d/, /l/, /s/, /a/, /o/, and /u/).

## 5. Discussion

This study introduced a methodology that enables the measurement of differences in the impact of dysarthria on different phonological classes, therefore reflecting its impact on different processes associated with speech production, including respiration, articulation, timing, and others. According to our findings, this methodology is suitable for the monitoring of speech deterioration in PD patients over time using recordings collected every month over several years. If the monitoring period is long enough, we could show which specific phonemes are systematically mispronounced over time (showing more degradation than others). This study introduced the use of phonological posteriors to assess the dysarthria level progression in a cohort of PD patients who were recorded three times in a time frame of three years (once per year). The ground truth was established as the values of the mFDA scale assigned by expert S&L therapists. Phonological models were based on a set with 18 Gated Recurrent Units (GRUs) that estimate the posterior probability of each phoneme to be “correctly pronounced” according to a set with 18 phonological classes. A total of 18 models, one per phonological class, were created per patient after analyzing the recordings of each session (three in total). Eighteen classification models (one per phonological class) for distinguishing between PD and HC subjects based on phonological posteriors were trained with the PC-GITA corpus. These 18 models were used to test the three recording sessions per speaker in the longitudinal analysis. No further optimization was performed for these classification models, i.e., the longitudinal corpus was used as a test set, representing a realistic scenario in which a patient comes to the clinic and requests to be evaluated to know whether his/her dysarthria level is progressing or not. The deterioration of “pronunciation correctness” was measured as the distance of each sample to the separating hyperplane created with the classifier. The mFDA labelling process showed that not all patients exhibit dysarthria level progression. This was validated in the first experiment. Afterward, we focused on evaluating only those patients who showed dysarthria level progression according to the mFDA. This filtering resulted in a subset of seven patients. When analyzing the recordings of those patients in session 1 vs. session 3, it was possible to observe specific patterns that might explain the progression of dysarthria. Specifically, strident, dental, pause, back, and continuant phonological classes arise as the ones that explain such a progression, which means that specific phonemes like /t/, /d/, /l/, /s/, /a/, /o/, and /u/ can be directly associated with this phenomenon.

Other studies in the literature have addressed the topic of the automatic evaluation and monitoring of PD symptoms considering speech-related biomarkers. Spectral-based analysis, articulation, and voice quality measures are among the most common [30], while respiration emerges as a biomarker due to its close relation with speech production [31]. Syllable counting, duration, and pause ratio have also been considered [32]. According to a review [33], phonation, articulation, and prosody features are found in most of the existing works. This paper introduces a new aspect of speech that is based on measuring how “correctly” the phonemes are being pronounced. According to our literature review, this is the first time this speech aspect has been used to evaluate the progression of dysarthic speech symptoms in PD patients.

Although specific phonemes showed certain patterns in our experiments, it is important to stress that this work set out to study the progression of PD over time being objectively evidenced in speech patterns. Such patterns allowed us to observe dysarthria progression from a broad perspective, rather than detecting specific patterns in specific phonemes with an association to PD symptoms. We believe that these findings provide objective evidence about possible abnormalities in broader speech-related processes like respiration, therefore contributing to a better understanding of the relationship between speech production patterns and other speech-related processes affected when suffering from PD.

**Limitations:** The small sample size considered in the longitudinal evaluations constitutes a clear limitation for this study. In fact, one-half of the cohort showed “improvement” in their mFDA score, and this phenomenon was not addressed in the paper. This could be a result of the fact that not every PD patient shows dysarthria, but it could also be because the scale is not sensitive enough. Further investigation is necessary to clarify this aspect. Additionally, this reduced sample size also limited our exploration of possible differentiating patterns between male and female patients. Therefore, increasing the number of participants would make the observations reported in this paper stronger, resulting in models that are more generalizable and suitable to the establishment of further discussion regarding specific gender-related disease behaviour. Additionally, this work has not addressed the problem of changing acoustic conditions over time, which constitutes a challenge in speech processing, especially when recordings are collected in at-home conditions.

## Figures and Tables

**Table 1:** Demographic and clinical information of the PC-GITA corpus. Values reported as mean ± standard deviation. (M): male; (F): female.

Variable	PD (M)	HC (M)	PD (F)	HC (F)
Number of subjects	25	25	25	25
Age (years) *	61.3 ± 11	60.5 ± 12	60.7 ± 7	61.4 ± 7
Age range (years)	33–81	31–86	49–75	49–76
Years since diagnosis	8.7 ± 6		12.6 ± 12	
MDS-UPDRS-III	37.8 ± 22		37.6 ± 14	
Speech item (MDS-UPDRS-III)	1.4 ± 0.9		1.3 ± 0.8	

* *p*-value = 0.77, calculated through a *t* test.

**Table 2:** Demographic and clinical information of the longitudinal corpus. [F/M]: female/male. Values reported as mean ± standard deviation.

Longitudinal Test Set			
	Session 1	Session 2	Session 3
Gender [F/M]	7/9	7/9	7/9
Age (years) [F/M]	63.2 ± 7.3/63.0 ± 9.2	64.2 ± 7.3/64.1 ± 9.4	65.7 ± 7.6/65.2 ± 9.2
Range of age [F/M]	52–71/48–79	53–73/50–81	54–75/52–82
Range of mFDA [F/M]	17–31/17–40	16–32/18–30	13–31/20–34
mFDA [F/M]	23.4 ± 5.7/28.7 ± 6.8	24.3 ± 6.0/22.9 ± 4.0	20.4 ± 6.0/26.7 ± 4.4

**Table 3:** mFDA per patient in the longitudinal corpus. Patients who showed dysarthria level progression between sessions 1 and 3 are highlighted in bold.

mFDA Values			
Patient	Session 1	Session 2	Session 3
**1**	**31**	**32**	**31**
**2**	**17**	**17**	**21**
**3**	**24**	**19**	**27**
**4**	**17**	**19**	**26**
5	25	18	20
**6**	**31**	**22**	**34**
7	28	29	16
8	17	26	17
**9**	**27**	**30**	**29**
10	19	23	13
11	25	27	25
12	36	24	30
13	40	22	25
14	31	27	21
**15**	**27**	**25**	**28**
16	27	16	20

**Table 4:** mFDA information of the longitudinal corpus and *PC-GITA* corpus. [F/M]: female/male. Values reported as mean ± standard deviation.

Longitudinal Corpus			
	Session 1	Session 2	Session 3
Gender [F/M]	7/9	7/9	7/9
mFDA [F/M]	23.4 ± 5.7/28.7 ± 6.8	24.3 ± 6.0/22.9 ± 4.0	20.4 ± 6.0/26.7 ± 4.4

**Table 5:** Phonological classes and their corresponding phonemes. * It is not a phoneme, but it was the way we used to code it in our algorithms.

#	Phonological Class	List of Phonemes
1	Vocalic	/a/, /e/, /i/, /o/, /u/
2	Consonantal	/b/, /tS/, /d/, /f/, /g/, /x/, /k/, /l/, /L/, /m/, /n/, /p/, /R/, /r/, /s/, /t/
3	Back	/a/, /o/, /u/
4	Anterior	/e/, /i/
5	Open	/a/, /e/, /o/
6	Close	/i/, /u/
7	Nasal	/m/, /n/
8	Stop	/p/, /b/, /t/, /k/, /g/, /tS/, /d/
9	Continuant	/f/, /b/, /tS/, /d/, /s/, /g/, /L/, /x/
10	Lateral	/l/
11	Flap	/R/
12	Trill	/r/
13	Voiced	/a/, /e/, /i/, /o/, /u/, /b/, /d/, /l/, /m/, /n/, /r/, /g/, /L/
14	Strident	/f/, /s/, /tS/
15	Labial	/m/, /p/, /b/, /f/
16	Dental	/t/, /d/
17	Velar	/k/, /g/, /x/
18	Silence	/sil/ *

**Table 6:** Results obtained within the context of the PC-GITA corpus. Upper part indicates results from the monologues while the bottom part includes results from read texts. AUC: area under the ROC curve. Lin: linear. RBF: Gaussian. Sig: sigmoid. *C*: penalty parameter of SVM. γ: RBF kernel bandwidth. η: Sigmoid offset. Values are reported as mean ± standard deviation. The last three columns indicate optimal values of hyper-parameters found within PC-GITA during training and used within the longitudinal corpus during test.

**Monologue**									
**Class**	**Accuracy**	**Sensitivity**	**Specificity**	**F1**	**AUC**	**Kernel**	C	γ	η
Vocalic	0.67 ± 0.17	0.74 ± 0.18	0.60 ± 0.25	0.69 ± 0.15	0.67 ± 0.17	RBF	0.7010	0.1467	-
Consonantal	0.64 ± 0.12	0.68 ± 0.18	0.60 ± 0.23	0.65 ± 0.10	0.64 ± 0.12	Lin	0.2196	-	-
**Back**	**0.75 ± 0.10**	**0.88 ± 0.13**	**0.62 ± 0.16**	**0.78 ± 0.09**	**0.75 ± 0.1**	**RBF**	5.2902	0.0950	-
Anterior	0.66 ± 0.16	0.68 ± 0.20	0.64 ± 0.19	0.66 ± 0.17	0.66 ± 0.17	RBF	494.9708	0.0010	-
**Open**	**0.70 ± 0.10**	**0.70 ± 0.18**	**0.70 ± 0.18**	**0.69 ± 0.13**	**0.70 ± 0.10**	**RBF**	34.3521	0.0274	-
Close	0.57 ± 0.20	0.50 ± 0.18	0.64 ± 0.33	0.54 ± 0.19	0.57 ± 0.20	Lin	0.0103	-	-
Nasal	0.59 ± 0.13	0.60 ± 0.17	0.58 ± 0.26	0.59 ± 0.14	0.59 ± 0.13	Lin	0.0463	-	-
Stop	0.60 ± 0.14	0.64 ± 0.12	0.56 ± 0.28	0.62 ± 0.11	0.60 ± 0.14	RBF	0.5966	0.0207	-
Continuant	0.61 ± 0.10	0.64 ± 0.17	0.58 ± 0.24	0.61 ± 0.10	0.61 ± 0.10	RBF	18,229.7583	6.0498	-
**Lateral**	**0.72 ± 0.14**	**0.74 ± 0.18**	**0.70 ± 0.22**	**0.72 ± 0.14**	**0.72 ± 0.14**	**Lin**	0.0408	-	-
Flap	0.69 ± 0.09	0.68 ± 0.24	0.70 ± 0.18	0.66 ± 0.15	0.69 ± 0.09	Sig	0.1602	2.5016	0.6104
Trill	0.64 ± 0.14	0.66 ± 0.15	0.62 ± 0.16	0.65 ± 0.14	0.64 ± 0.14	RBF	1850.1336	3.7337	0.0000
Voiced	0.69 ± 0.13	0.70 ± 0.20	0.68 ± 0.27	0.69 ± 0.14	0.69 ± 0.14	Sig	28.3304	0.0494	0.0000
Strident	0.64 ± 0.11	0.74 ± 0.22	0.54 ± 0.18	0.66 ± 0.13	0.64 ± 0.11	RBF	980.0120	0.0003	-
Labial	0.69 ± 0.09	0.80 ± 0.15	0.58 ± 0.22	0.72 ± 0.08	0.69 ± 0.09	Lin	149.1163	-	-
Dental	0.63 ± 0.09	0.48 ± 0.13	0.78 ± 0.21	0.56 ± 0.08	0.63 ± 0.09	Sig	0.2598	0.9179	3.1037
Velar	0.64 ± 0.10	0.74 ± 0.18	0.54 ± 0.28	0.67 ± 0.08	0.64 ± 0.10	Lin	0.4694	-	-
Pause	0.63 ± 0.12	0.64 ± 0.25	0.62 ± 0.21	0.62 ± 0.15	0.63 ± 0.12	Sig	3614.6284	0.0070	0.0650
Mean	0.65	0.68	0.62	0.65	0.65	Na	Na	Na	Na
**Read Text**									
**Class**	**Accuracy**	**Sensitivity**	**Specificity**	**F1**	**AUC**	**Kernel**	C	γ	η
Vocalic	0.65 ± 0.12	0.54 ± 0.18	0.76 ± 0.30	0.60 ± 0.14	0.65 ± 0.13	Lin	0.1503	-	-
**Consonantal**	**0.71 ± 0.14**	**0.74 ± 0.15**	**0.68 ± 0.28**	**0.72 ± 0.10**	**0.71 ± 0.14**	**RBF**	0.9350	0.1608	-
**Back**	**0.81 ± 0.11**	**0.76 ± 0.15**	**0.86 ± 0.18**	**0.80 ± 0.11**	**0.81 ± 0.11**	**RBF**	6969.0972	0.0002	-
Anterior	0.66 ± 0.17	0.66 ± 0.28	0.66 ± 0.18	0.63 ± 0.22	0.66 ± 0.17	Lin	0.4608	-	-
**Open**	**0.70 ± 0.11**	**0.60 ± 0.20**	**0.80 ± 0.22**	**0.65 ± 0.13**	**0.70 ± 0.12**	**Lin**	0.1831	-	-
Close	0.67 ± 0.09	0.66 ± 0.12	0.68 ± 0.20	0.66 ± 0.08	0.67 ± 0.09	Lin	1.2850	-	-
Nasal	0.59 ± 0.11	0.54 ± 0.22	0.64 ± 0.21	0.55 ± 0.14	0.59 ± 0.11	RBF	114.9516	0.0093	-
Stop	0.67 ± 0.11	0.50 ± 0.18	0.84 ± 0.19	0.59 ± 0.17	0.67 ± 0.11	Lin	0.0231	-	-
Continuant	0.66 ± 0.08	0.38 ± 0.14	0.94 ± 0.09	0.51 ± 0.14	0.66 ± 0.08	RBF	0.7602	0.0160	-
Lateral	0.62 ± 0.12	0.64 ± 0.12	0.60 ± 0.22	0.63 ± 0.11	0.62 ± 0.12	Lin	0.9104	-	-
**Flap**	**0.70 ± 0.13**	**0.64 ± 0.23**	**0.76 ± 0.19**	**0.66 ± 0.18**	**0.70 ± 0.13**	**Sig**	0.0784	0.9477	3.8600
Trill	0.57 ± 0.14	0.54 ± 0.24	0.60 ± 0.24	0.53 ± 0.21	0.57 ± 0.14	Sig	20.9498	14,640.8440	0.0000
Voiced	0.67 ± 0.15	0.60 ± 0.13	0.74 ± 0.22	0.65 ± 0.14	0.67 ± 0.15	RBF	2983.3609	0.0007	-
Strident	0.64 ± 0.11	0.38 ± 0.19	0.90 ± 0.13	0.49 ± 0.22	0.64 ± 0.11	Sig	0.1486	0.1034	0.0560
**Labial**	**0.71 ± 0.14**	**0.74 ± 0.15**	**0.68 ± 0.22**	**0.72 ± 0.13**	**0.71 ± 0.14**	**Sig**	47.1880	0.0146	0.0496
Dental	0.65 ± 0.13	0.48 ± 0.22	0.82 ± 0.19	0.55 ± 0.23	0.65 ± 0.13	RBF	2.0605	0.4366	-
Velar	0.58 ± 0.13	0.52 ± 0.22	0.64 ± 0.17	0.53 ± 0.18	0.58 ± 0.13	RBF	9232.2919	0.0013	-
Pause	0.65 ± 0.18	0.58 ± 0.17	0.72 ± 0.32	0.63 ± 0.16	0.65 ± 0.18	Lin	0.0438	-	-
Mean	0.66	0.58	0.74	0.61	0.66	Na	Na	Na	Na

**Table 7:** Area of the radar plots generated with the accuracy scores obtained from the longitudinal recordings.

Sessions	Monologue	Read Text
1	0.6027	0.6285
2	0.6736	0.6553
3	0.7528	0.7688

**Table 8:** Area of radar plots generated with the accuracy scores obtained from the set of seven patients who showed dysarthria level progression between session 1 and session 3 according to the mFDA values.

Sessions	Monologue	Read Text
1	0.6426	0.6695
3	0.6452	0.8858

## Data Availability

Due to ethical constraints, the longitudinal corpus used in this study is not publicly available. That of the PC-GITA group can be provided by the corresponding author upon request.

## References

[1] Goldman J.G., Volpe D., Ellis T.D., Hirsch M.A., Johnson J., Wood J., Aragon A., Biundo R., Di Rocco A., Kasman G.S. (2024). Delivering multidisciplinary rehabilitation care in Parkinson’s disease: An international consensus statement. J. Park. Dis..

[2] Naranjo L., Pérez C.J., Campos-Roca Y. (2021). Monitoring Parkinson’s disease progression based on recorded speech with missing ordinal responses and replicated covariates. Comput. Biol. Med..

[3] Bugalho P., Ladeira F., Barbosa R., Marto J.P., Borbinha C., da Conceição L., Salavisa M., Saraiva M., Meira B., Fernandes M. (2021). Progression in parkinson’s disease: Variation in motor and non-motor symptoms severity and predictors of decline in cognition, motor function, disability, and health-related quality of life as assessed by two different methods. Mov. Disord. Clin. Pract..

[4] Tzallas A.T., Tsipouras M.G., Rigas G., Tsalikakis D.G., Karvounis E.C., Chondrogiorgi M., Psomadellis F., Cancela J., Pastorino M., Waldmeyer M.T.A. (2014). PERFORM: A system for monitoring, assessment and management of patients with Parkinson’s disease. Sensors.

[5] Rong S., Xu G., Liu B., Sun Y., Snetselaar L.G., Wallace R.B., Li B., Liao J., Bao W. (2021). Trends in mortality from Parkinson disease in the United States, 1999–2019. Neurology.

[6] Orozco-Arroyave J., Vásquez-Correa J., Klumpp P., Pérez-Toro P.A., Escobar-Grisales D., Roth N., Ríos-Urrego C.D., Strauss M., Carvajal-Castaño H.A., Bayerl S. (2020). Apkinson: The smartphone application for telemonitoring Parkinson’s patients through speech, gait, and hands movement. Neurodegener. Dis. Manag..

[7] Trail M., Fox C., Ramig L.O., Sapir S., Howard J., Lai E.C. (2005). Speech treatment for Parkinson’s disease. NeuroRehabilitation.

[8] Wodzinski M., Skalski A., Hemmerling D., Orozco-Arroyave J.R., Nöth E. (2019). Deep learning approach to Parkinson’s disease detection using voice recordings and convolutional neural network dedicated to image classification. Proceedings of the 2019 41st Annual International Conference of the IEEE Engineering in Medicine and Biology Society (EMBC).

[9] Pradeep P., Kamalakannan J. (2024). Comprehensive review of literature on Parkinson’s disease diagnosis. Comput. Biol. Chem..

[10] Hlavnicka J., Cmejla R., Tykalová T., Šonka K., Růžička E., Rusz J. (2017). Automated analysis of connected speech reveals early biomarkers of Parkinson’s disease in patients with rapid eye movement sleep behaviour disorder. Sci. Rep..

[11] Roland V., Huet K., Harmegnies B., Piccaluga M., Verhaegen C., Delvaux V. (2023). Vowel production: A potential speech biomarker for early detection of dysarthria in Parkinson’s disease. Front. Psychol..

[12] Lansford K., Liss J. (2014). Vowel acoustics in dysarthria: Speech disorder diagnosis and classification. J. Speech Lang. Hear. Res..

[13] Di Pietro D.A., Olivares A., Comini L., Vezzadini G., Luisa A., Petrolati A., Boccola S., Boccali E., Pasotti M., Danna L. (2022). Voice alterations, dysarthria, and respiratory derangements in patients with Parkinson’s disease. J. Speech Lang. Hear. Res..

[14] Moya-Galé G., Goudarzi A., Bayés À., McAuliffe M., Bulté B., Levy E. (2018). The effects of intensive speech treatment on conversational intelligibility in Spanish speakers with Parkinson’s disease. Am. J. Speech-Lang. Pathol..

[15] Tsanas A., Little M.A., McSharry P.E., Spielman J., Ramig L.O. (2012). Novel speech signal processing algorithms for high-accuracy classification of Parkinson’s disease. IEEE Trans. Biomed. Eng..

[16] Orozco-Arroyave J.R. (2015). Analysis of Speech of People with Parkinson’s Disease.

[17] Orozco-Arroyave J.R., Vásquez-Correa J.C., Vargas-Bonilla J.F., Arora R., Dehak N., Nidadavolu P., Christensen H., Rudzicz F., Yancheva M., Chinaei H. (2018). NeuroSpeech: An open-source software for Parkinson’s speech analysis. Digit. Signal Process..

[18] Vásquez-Correa J., Klumpp P., Orozco-Arroyave J., Nöth E. (2019). Phonet: A Tool Based on Gated Recurrent Neural Networks to Extract Phonological Posteriors from Speech. Proceedings of the Interspeech.

[19] Klumpp P., Arias-Vergara T., Vásquez-Correa J., Pérez-Toro P., Orozco-Arroyave J., Batliner A., Nöth E. (2021). The Phonetic Footprint of Parkinson’s Disease. Comput. Speech Lang..

[20] Moro-Velazquez L., Gomez-Garcia J.A., Godino-Llorente J.I., Grandas-Perez F., Shattuck-Hufnagel S., Yagüe-Jimenez V., Dehak N. (2019). Phonetic relevance and phonemic grouping of speech in the automatic detection of Parkinson’s Disease. Sci. Rep..

[21] Liu Y., Reddy M.K., Penttilä N., Ihalainen T., Alku P., Räsänen O. (2022). Automatic assessment of Parkinson’s disease using speech representations of phonation and articulation. IEEE/ACM Trans. Audio Speech Lang. Process..

[22] Eyben F., Scherer K., Schuller B., Sundberg J., Andre E., Busso C., Devillers L., Epps J., Laukka P., Narayanan S. (2016). The Geneva Minimalistic Acoustic Parameter Set (GeMAPS) for Voice Research and Affective Computing. IEEE Trans. Affect. Comput..

[23] Escobar-Grisales D., Arias-Vergara T., Rios-Urrego C.D., Nöth E., García A.M., Orozco-Arroyave J.R. (2023). An automatic multimodal approach to analyze linguistic and acoustic cues on Parkinson’s disease patients. Proceedings of the Interspeech.

[24] Skodda S., Grönheit W., Mancinelli N., Schlegel U. (2013). Progression of Voice and Speech Impairment in the Course of Parkinson’s Disease: A Longitudinal Study. Park. Dis..

[25] Arias-Vergara T., Vásquez-Correa J., Orozco-Arroyave J., Nöth E. (2018). Speaker models for monitoring Parkinson’s disease progression considering different communication channels and acoustic conditions. Speech Commun..

[26] Garcia A.M., Arias-Vergara T., Vásquez-Correa J., Nöth E., Schuster M., Welch A., Bocanegra Y., Baena A., Orozco-Arroyave J. (2021). Cognitive determinants of dysarthria in Parkinson’s disease: An automated machine learning approach. Mov. Disord..

[27] Orozco-Arroyave J.R., Arias-Londoño J.D., Vargas-Bonilla J.F., Gonzalez-Rátiva M.C., Nöth E. New Spanish speech corpus database for the analysis of people suffering from Parkinson’s disease. Proceedings of the LREC.

[28] Goetz C.G., Tilley B.C., Shaftman S.R., Stebbins G.T., Fahn S., Martinez-Martin P., Poewe W., Sampaio C., Stern M.B., Dodel R. (2008). Movement Disorder Society-sponsored revision of the Unified Parkinson’s Disease Rating Scale (MDS-UPDRS): Scale presentation and clinimetric testing results. Mov. Disord..

[29] Vásquez-Correa J.C., Orozco-Arroyave J., Bocklet T., Nöth E. (2018). Towards an automatic evaluation of the dysarthria level of patients with Parkinson’s disease. J. Commun. Disord..

[30] Brinkman J. (2014). A Longitudinal Study of Parkinsonian Speech Characteristics. Master’s Thesis.

[31] Darling-White M., Anspach Z., Huber J. (2022). Longitudinal Effects of Parkinson’s Disease on Speech Breathing During an Extemporaneous Connected Speech Task. J. Speech Lang. Hear. Res..

[32] dos Santos V.B., Zardin F.V., Rothe-Neves R., Olchik M.R. (2025). Does speech in patients with different Parkinson’s disease subtypes decline over time?. Clin. Park. Relat. Disord..

[33] Wright H., Aharonson V. (2025). Vocal Feature Changes for Monitoring Parkinson’s Disease Progression—A Systematic Review. Brain Sci..

